# Evaluating Quality of Life Changes over 12 Months Among Opiate Users from Romania and Associated Worsening Factors

**DOI:** 10.3390/life14101336

**Published:** 2024-10-21

**Authors:** Melania Lavinia Bratu, Dorel Sandesc, Teodora Anghel, Felix Bratosin, Silviu Valentin Vlad, Artiom Terzi, Caius Glad Streian

**Affiliations:** 1 Center for Neuropsychology and Behavioral Medicine, Department of Psychology, Faculty of General Medicine, “Victor Babes” University of Medicine and Pharmacy Timisoara, Eftimie Murgu Square 2, 300041 Timisoara, Romania; bratu.lavinia@umft.ro (M.L.B.); anghel.teodora@umft.ro (T.A.); 2 Doctoral School, “Victor Babes” University of Medicine and Pharmacy Timisoara, Eftimie Murgu Square 2, 300041 Timisoara, Romania; 3 Center for Cognitive Research in Neuropsychiatric Pathology, Department of Neurosciences, “Victor Babes” University of Medicine and Pharmacy Timisoara, Eftimie Murgu Square 2, 300041 Timisoara, Romania; 4 Department of Anesthesia and Intensive Care, “Victor Babes” University of Medicine and Pharmacy Timisoara, Eftimie Murgu Square 2, 300041 Timisoara, Romania; sandesc.dorel@umft.ro; 5 Methodological and Infectious Diseases Research Center, Department of Infectious Diseases, “Victor Babes” University of Medicine and Pharmacy Timisoara, Eftimie Murgu Square 2, 300041 Timisoara, Romania; felix.bratosin@umft.ro; 6 Department of Surgery, Faculty of Medicine, University of Oradea, 410073 Oradea, Romania; 7 Department of General Medicine, “Nicolae Testemitanu” State University of Medicine and Pharmacy, Stefan cel Mare si Sfant Boulevard 165, 2004 Chisinau, Moldova; artiomterzimd@gmail.com; 8 Department of Cardiac Surgery, “Victor Babes” University of Medicine and Pharmacy Timisoara, 300041 Timisoara, Romania; streian.caius@umft.ro

**Keywords:** quality of life, psychiatry, opiate, drug abuse

## Abstract

This study aimed to evaluate the changes in quality of life (QoL) over a 12-month period among opiate users in Romania, identifying factors that contribute to a worsening of their condition. By examining these dynamics, the research intended to inform targeted interventions and support mechanisms to mitigate the negative outcomes associated with opiate use. Conducted as a longitudinal cohort analysis, this study enrolled 74 participants diagnosed with opioid use disorders from multiple healthcare settings in Romania. The WHOQOL-BREF instrument was utilized to assess QoL, with data collection spanning from 1 January 2023 to 31 December 2023. Ethical compliance with the Declaration of Helsinki was maintained, and participants provided informed consent. Statistical analysis was performed using SPSS, focusing on the impact of demographic and behavioral variables on QoL. Over the 12-month period, significant improvements were observed in all QoL domains: physical (51.68 to 58.39, *p* < 0.001), psychological (49.34 to 55.32, *p* < 0.001), social (46.21 to 53.66, *p* < 0.001), and environmental (47.85 to 54.17, *p* < 0.001). Methadone compliance significantly influenced positive outcomes across all domains. Compliant participants exhibited higher mean scores compared to non-compliant users, with respective increases in the physical domain from 52.47 to 60.21 (*p* < 0.001), psychological from 50.93 to 58.32 (*p* < 0.001), social from 48.36 to 57.14 (*p* < 0.001), and environmental from 47.82 to 55.79 (*p* < 0.001). Additionally, education showed a protective effect, particularly enhancing environmental QoL (estimate = 0.33, *p* = 0.013). Methadone compliance and higher education levels were identified as significant predictors of improved QoL among opiate users, demonstrating the critical importance of adherence to treatment protocols and the supportive role of education in enhancing life quality. These findings highlight the necessity for integrated treatment programs and educational interventions to improve the well-being of individuals battling opioid addiction, advocating for policy enhancements and supportive measures tailored to this demographic.

## 1. Introduction

The escalating global crisis of opiate use and its impact on public health has been a topic of significant concern over the past decade. Opiates, which include both prescription pain relievers and illegal drugs like heroin, are linked to a high risk of addiction and overdose [1,2]. According to the World Drug Report 2023 by the United Nations Office on Drugs and Crime (UNODC), approximately 58 million people worldwide used opioids in 2022, a figure that underscores the pervasive nature of this issue. This increasing trend is particularly alarming given the heightened risk of mortality; globally, opioids were responsible for two-thirds of the 500,000 drug-related deaths reported in 2021 [3,4,5,6].

In Europe, the situation mirrors the global crisis. The European Monitoring Centre for Drugs and Drug Addiction (EMCDDA) highlighted in its 2023 report that opioids continue to be a major driver of drug-related health consequences, including deaths [7,8]. In 2022, opioids accounted for over 75% of drug overdose deaths in Europe, indicating a sustained public health challenge [9,10]. The reports also emphasized the growing problem of synthetic opioids, which are more potent and pose a greater overdose risk than naturally derived opioids [11,12].

In Romania, the dynamics of opiate use present unique challenges. Although the country has historically reported lower rates of drug use compared to Western Europe, recent trends indicate a rise in opioid consumption, both in terms of illegal heroin and misuse of prescription opioids [13,14]. This shift raises concerns about the adequacy of the existing healthcare and support systems to manage and mitigate the consequences of opioid addiction.

The abuse of opioids, including both prescription medications and illicit substances, is a key driver of the current crisis, leading to widespread addiction and overdose deaths [15]. The misuse of prescription opioids in particular has contributed significantly to the escalation of opioid dependency, often serving as a gateway to the use of more potent illicit drugs such as heroin and synthetic opioids. The opioid crisis, though longstanding in the U.S., has more recently become a significant concern in Europe and Romania as well, where rising cases of opioid misuse, abuse, and medication errors have been reported [13]. Recent studies highlight the increasing incidence of adverse events linked to opioids like fentanyl, morphine, and oxycodone, particularly in Europe [16]. These findings underscore the need for greater vigilance and preventative measures in opioid prescribing and use across the continent. As the crisis evolves, both public health systems and regulatory bodies in Europe must address these issues to mitigate opioid-related harm. Integrating such data enhances the understanding of opioid misuse trends and offers valuable context for this study’s focus on opioid use in Romania.

The adverse effects of opiate use extend beyond the risk of overdose. Chronic use of opiates significantly diminishes quality of life (QoL), impacting physical health, mental well-being, social relationships, and economic productivity [17]. Individuals addicted to opioids often experience a range of debilitating health issues, including an increased risk of infectious diseases, mental health disorders, and sustained injuries due to drug-seeking behaviors [18]. Moreover, opioid misuse and abuse lead to numerous adverse events beyond death, including dependence and withdrawal, even at therapeutic doses. Pharmacovigilance data, such as those from EudraVigilance and FDA reports, highlight the underreporting of these issues. Studies reveal gaps in adverse event reporting, emphasizing the need for better monitoring of opioid-related harm [19,20].

Research into the long-term impact of opioid use on quality of life is still evolving, particularly in Eastern European regions where research is very scarce to non-existent. Studies from other regions have shown that continuous opioid use is associated with a progressive decline in health-related quality of life, compounded by factors such as socioeconomic status, comorbidities, and the availability of support services [21]. However, data from Eastern Europe, and Romania specifically, remain sparse, highlighting a critical gap in the literature.

This study aims to evaluate the changes in quality of life over a 12-month period among opiate users in Romania, identifying the primary factors that contribute to a worsening of their condition. By understanding these dynamics, the study seeks to inform targeted interventions and support mechanisms that can be implemented to alleviate the negative outcomes associated with opiate use in this demographic.

## 2. Materials and Methods

### 2.1. Study Design and Ethical Considerations

This study was designed as a longitudinal cohort analysis, focusing on individuals with opioid use disorders across multiple healthcare settings within Romania. The primary sites for data collection were the clinics affiliated with the Victor Babes University of Medicine and Pharmacy from Timisoara. The study period spanned from 1 January 2023 to 31 December 2023, bringing together specialists in addiction medicine, psychiatry, and public health to ensure a comprehensive approach to examining the impact of opioid use on quality of life.

Adhering to rigorous ethical standards, the research was conducted in full compliance with the Declaration of Helsinki—Ethical Principles for Medical Research Involving Human Subjects. Participation in the study was entirely voluntary, with all participants providing informed consent after receiving detailed information about the purpose of the study, the procedures involved, the confidentiality of their data, and their right to withdraw at any time without consequence to their medical care or personal integrity.

To ensure ethical integrity and safeguard participant welfare, the study received approval from the Ethics Committee of the “Victor Babes” Central Military Emergency University Hospital and the affiliated “Victor Babeș” University of Medicine and Pharmacy from Timisoara (Approval code 2429 from 10 January 2022). This approval encompassed all aspects of the study, including data collection methods, participant recruitment strategies, and the handling of sensitive information. In the preparation of this manuscript, ChatGPT (OpenAI, San Francisco, CA, USA) was utilized to assist with English grammar and spelling correction, ensuring linguistic accuracy and clarity.

### 2.2. Inclusion and Exclusion Criteria

The inclusion criteria for this study were defined to ensure a well-defined study population, specifically targeted at individuals affected by opioid use disorders. The criteria included the following: (1) Diagnosis of opioid use disorder: Participants must have a formal diagnosis of opioid use disorder based on the DSM-5 (Diagnostic and Statistical Manual of Mental Disorders, Fifth Edition) criteria [22], ensuring the study population was homogeneously affected by this condition; (2) Treatment status: Participants must be either currently receiving treatment or in a recovery program for opioid use within the study’s designated timeframe, allowing for a consistent assessment of quality of life impacts in a recovery-oriented environment; (3) Age: Individuals aged 18 years and above were included, as this reflects the legal adulthood age in Romania, ensuring participants can provide informed consent; (4) Consent: Ability and willingness to provide informed consent for participation in the study, upholding ethical standards; and (5) Residency: Participants must be residents of Romania to reflect the geographic focus of the study and ensure accessibility for follow-up assessments.

The exclusion criteria were established to isolate the effects of opioid use on quality of life from other confounding factors. These criteria comprised the following: (1) Severe cognitive impairment: Individuals with severe cognitive impairment or neurological conditions significantly affecting comprehension or communication are excluded to ensure the reliability of survey responses; (2) Concurrent severe psychiatric disorders: Patients with severe psychiatric disorders other than opioid use disorder, which could independently affect their quality of life significantly, were excluded. This maintained the study’s focus on the impacts of opioid use; (3) Critical physical illness: Patients with critical physical illnesses that could independently and significantly affect quality of life measurements were excluded to avoid confounding the effects of opioid use with other health conditions; (4) Lack of consent: Individuals who were unable or unwilling to provide informed consent were excluded, adhering to the ethical principles governing human research; (5) Language barrier: Patients who were unable to understand or communicate in Romanian, the language in which the study assessments and interventions were conducted, were excluded to ensure participants fully comprehended the content and provided valid responses.

The exclusion criteria for this study also comprised individuals with severe cognitive impairment, assessed using the Mini-Mental State Examination (MMSE) with a cutoff score of ≤24, which ensured exclusion of patients with conditions like advanced dementia that could hinder comprehension or communication. Additionally, patients with concurrent severe psychiatric disorders, such as uncontrolled psychosis or bipolar disorder, based on the DSM-5 criteria, were excluded to maintain focus on the impacts of opioid use. Lastly, those with critical physical illnesses, such as advanced cancer or end-stage organ failure, which could significantly confound quality of life assessments, were also excluded.

A control group of 50 individuals was selected to evaluate the role of comorbidity and social factors on quality of life. The control group consisted of healthy individuals without opioid use disorder or major comorbidities, matched by age and gender to the study participants. These individuals were recruited from the general population through community health clinics and underwent the same WHOQOL-BREF survey as the study groups. The purpose of including the control group was to provide a baseline for comparing quality of life across different domains, helping to assess the specific impact of opioid use, methadone compliance, and associated comorbidities.

### 2.3. WHOQOL

In this longitudinal study, we employed the WHOQOL-BREF instrument [23] to assess the quality of life among individuals with opioid use disorders in Romania. The WHOQOL-BREF is a shorter version of the comprehensive WHOQOL-100 assessment tool and has been validated for use in multiple languages, including Romanian. This validation and its widespread international use make it an appropriate tool for assessing diverse aspects of quality of life in a culturally sensitive manner.

The WHOQOL-BREF instrument was administered in its Romanian-translated form to assure cultural relevance and clarity of comprehension. Participants were asked to rate each question on a five-point Likert scale, ranging from 1 (not at all) to 5 (an extreme amount). The scoring for the WHOQOL-BREF followed the standard guidelines provided by the World Health Organization, which involves transforming raw scores into a scale ranging from 0 to 100 for easier interpretation and comparison. The final scores in the domains of physical health, psychological health, social relationships, and environment were calculated by aggregating and standardizing the item scores within each respective domain, providing a comprehensive overview of the quality of life across these key areas for each participant.

### 2.4. Statistical Analysis

A power analysis was conducted to determine the required sample size for detecting significant differences in quality of life over a 12-month period among opiate users. Using an effect size of 0.5 (medium effect), a power of 0.80, and a significance level (alpha) of 0.05, the minimum sample size needed was calculated to be 60 participants. This calculation aimed to ensure that the study had sufficient power to detect meaningful changes in quality of life, with a 95% confidence level and a 10% margin of error. Taking into account potential dropouts and incomplete data, an initial recruitment target of 100 participants was set, resulting in 74 participants completing the study period.

Data management and analysis were conducted utilizing the statistical software SPSS version 26.0 (SPSS Inc., Chicago, IL, USA). Continuous variables were represented as mean ± standard deviation (SD), while categorical variables were expressed in terms of frequencies and percentages. The Student’s *t*-test for comparing two means between the continuous data. Additional analyses using Shapiro–Wilk and Levene’s tests were performed. The Chi-square test was utilized for the categorical variables. A linear regression analysis was performed to identify the significant factors affecting the QoL in the target population. A *p*-value threshold of less than 0.05 was set for statistical significance.

## 3. Results

### Background Characteristics

A total of 74 opiate users were evaluated for various demographic and clinical characteristics. The average age of the participants was 35.27 years with a standard deviation of 9.43 years, indicating a middle-aged cohort with moderate age variability. The distribution across age groups showed a higher proportion of individuals in the 30–40 years range (39.19%), followed by those over 40 years (32.43%), and the youngest group, 18–29 years, constituted 28.38%. Regarding gender distribution, the majority were men (60.81%).

Educational background varied significantly among the participants: 33.78% had completed high school, making it the most common education level, followed by university graduates (21.62%), and middle school attendees (22.97%). The rates of comorbidities were notably high, with 28.38% reporting diabetes mellitus and 16.22% having hypertension, reflecting common health challenges among the cohort. Smoking and chronic alcohol use were reported in 63.51% and 51.35% of the participants, respectively (Table 1).

The longitudinal assessment of quality of life among opiate users in Romania, as measured by the WHOQOL-BREF survey, revealed statistically significant improvements across all QoL domains over a 12-month period. Initially, the physical domain was recorded at 51.68 with a standard deviation of 8.35, which progressively increased to 58.39 by the 12-month mark, with a corresponding *p*-value of less than 0.001, indicating a substantial improvement. Similarly, the psychological domain showed an upward trend from an initial mean of 49.34 to 55.32, with each subsequent measurement also demonstrating significant enhancement. The social and environmental domains followed comparable patterns, starting at 46.21 and 47.85, respectively, and reaching 53.66 and 54.17 by the end of the study period, both with *p*-values less than 0.001, reflecting significant positive changes. In contrast to the QoL domains, the methadone dosage administered to participants did not show a statistically significant change over the 12 months, starting at a mean of 65.74 mg and fluctuating slightly before ending at 62.58 mg, with a *p*-value of 0.406 (Table 2).

The results indicated that methadone compliance significantly influences quality of life across all measured domains. Specifically, compliant participants (*n* = 48) consistently exhibited higher mean scores in each domain compared to their non-compliant counterparts (*n* = 26). In the physical domain, compliant users scored an average of 60.21 with a standard deviation of 7.34, markedly higher than the 52.47 scored by non-compliant users, which also had a higher variation (SD = 8.56), reflected by a *p*-value of less than 0.001.

Similarly, substantial differences were observed in the psychological, social, and environmental domains, all favoring compliant individuals. Compliant users scored 58.32 in the psychological domain compared to 50.93 by non-compliant users; 57.14 vs. 48.36 in the social domain; and 55.79 vs. 47.82 in the environmental domain, each with a statistically significant *p*-value of less than 0.001. The individuals from the control group exhibited significantly better QoL scores, evidencing the impact that substance use has on life quality (Table 3).

Notably, the variable of methadone compliance versus noncompliance demonstrated a significant positive effect on physical quality of life, with an estimate of 3.17 and a highly significant *p*-value of less than 0.001. The confidence interval (1.67, 4.67) did not encompass zero, indicating a robust relationship. Conversely, most other demographic predictors did not show statistically significant effects. For instance, comparisons of age categories showed no significant differences with the 30-40 years group versus the 18–29 years group yielding an estimate of 0.86 and a *p*-value of 0.265. Similarly, the >40 years group showed a negative association with physical quality of life compared to the 18–29 years group, but this was not statistically significant (*p*-value of 0.082). Additionally, variables such as hypertension, diabetes mellitus, smoking, and chronic alcohol use also did not significantly affect the physical quality of life, with *p*-values well above the 0.05 threshold (Table 4).

It was observed that relationship status significantly impacted psychological well-being, with individuals who were married or in a relationship reporting higher quality of life scores than those who were single. Specifically, being married versus single was associated with a substantial improvement (estimate = 1.84, *p*-value = 0.021), and being in a relationship versus single also demonstrated a significant positive effect (estimate = 2.29, *p*-value = 0.007). Additionally, methadone compliance emerged as a significant positive factor, with compliant individuals showing a notably higher psychological quality of life score compared to non-compliant users (estimate = 2.16, *p*-value = 0.021), as seen in Table 5.

Education demonstrated a notably positive effect, with each additional level of education associated with an increase in the social quality of life score (estimate = 0.49, *p*-value = 0.001). Relationship status significantly influenced social well-being, particularly for those in a relationship versus single individuals, with an estimate of 2.14 and a *p*-value of 0.015, highlighting the beneficial impact of supportive intimate relationships on social quality of life. Additionally, methadone compliance was associated with an improvement in social quality of life (estimate = 1.60, *p*-value = 0.008), underscoring the importance of adherence to treatment in fostering better social integration and support among opiate users. In contrast, smoking was found to negatively impact social quality of life, with a significant decrease in social scores for smokers (estimate = −1.04, *p*-value = 0.026), as presented in Table 6.

The degree of education significantly contributed to higher quality of life scores, with each incremental level of education increasing the environmental quality of life score by 0.33 (*p*-value = 0.013). Methadone compliance was another significant positive predictor, with compliant individuals scoring higher by an average of 2.41 points compared to noncompliant ones (*p*-value = 0.002). In contrast, variables such as age, sex, and comorbid conditions like hypertension and diabetes did not show a significant direct effect on the environmental dimension of quality of life (Table 7).

## 4. Discussion

### 4.1. Literature Findings

The data from this study highlight significant improvements in quality of life (QoL) across all domains among opiate users over a 12-month period, particularly for those who are compliant with methadone treatment. The WHOQOL-BREF results show a consistent upward trend in physical, psychological, social, and environmental domains for both men and women, regardless of comorbidity status, suggesting that methadone compliance is associated with better QoL outcomes. Notably, individuals without comorbidities had slightly higher baseline and follow-up scores, reflecting the additional burden that comorbid conditions may impose on overall well-being.

The impact of comorbidities was evident across all domains, with participants with comorbidities consistently scoring lower than those without. This trend was observed in both men and women, emphasizing the role that additional health conditions play in moderating the benefits of methadone treatment. For example, men with no comorbidities showed the largest improvement in physical and psychological domains, while women with comorbidities demonstrated slower progress in these areas. These findings suggest that more tailored interventions addressing comorbidities may be necessary to optimize outcomes for this subgroup.

When comparing the compliant and uncompliant groups, a stark contrast emerges. Compliant individuals reported significantly higher QoL scores across all domains compared to uncompliant participants. This suggests that methadone adherence is a critical factor in improving the well-being of opiate users. Additionally, the comparison with the control group of healthy individuals provides valuable context. The control group consistently scored higher in all domains, underscoring the persistent QoL gap between opiate users, even those compliant with treatment, and the general population. However, the substantial improvement among compliant users demonstrates the potential of methadone programs in bridging this gap.

In a similar manner, the systematic review by Jeremy W. Bray et al. [24] and the secondary analysis of a clinical trial by Ali Jalali et al. [25] underscore the significance and underutilization of health-related quality of life (HRQoL) measures in opioid use disorder (OUD) treatments. Bray et al. found that out of 92 reviewed articles, only 17 used validated HRQoL measures, highlighting a gap in patient-centered outcomes in opioid treatment programs. Conversely, Jalali et al.’s study on 508 participants revealed two distinct subpopulations in HRQoL responses to pharmacotherapy—82.3% showed sustained improvements, while 17.7% exhibited transient gains, influenced by socioeconomic and psychosocial factors [25]. Both studies advocate for broader integration of HRQoL assessments to enhance personalized care and policy-making in OUD treatments.

Moreover, Aas et al. [26] conducted a nested prospective cohort study in Norway involving 609 OAT patients, finding that the baseline HRQoL (EQ-5D-5L index value 0.699, SD 0.250) was significantly lower than that of the general Norwegian population (0.848, SD 0.200). The study noted significant improvements in mobility and pain/discomfort by the 1-year follow-up, although overall HRQoL scores remained below national averages. Similarly, Nosyk et al. [27] analyzed data from two randomized controlled trials, revealing short-term HRQoL improvements across different OAT modalities, with initial benefits that diminished over time. Specifically, the effect sizes ranged from 0.039 in heroin users receiving buprenorphine/naloxone to 0.071 in prescription opioid users receiving methadone, indicating modest but tangible initial improvements in HRQoL. Both studies underscore the potential of OAT to enhance HRQoL among opioid-dependent individuals, though Aas et al.’s long-term follow-up suggests that achieving HRQoL comparable to the general population may require additional supportive measures beyond standard pharmacotherapy.

Whitehurst et al. [28] investigated the sensitivity of three HRQoL instruments (EQ-5D-3L, EQ-5D-5L, HUI3) among Canadian patients with prescription-type opioid use disorder, reporting similar magnitudes of change across the instruments, with no superior sensitivity to change demonstrated; the HUI3, for example, showed six of the ten highest area under the ROC curve estimates, indicating modest discriminative ability. In contrast, Cheng et al. [29] assessed HRQoL in Australian people who inject drugs, finding that employed participants had significantly higher HRQoL scores with a mean EQ-5D-3L score of 0.83 and EQ-VAS score of 77, compared to the overall mean EQ-5D-3L score of 0.67 and EQ-VAS score of 62 for the cohort. These findings highlight the complex interplay between treatment modalities, socioeconomic status, and HRQoL outcomes in opioid-dependent populations.

The studies by Martin Kåberg et al. [30] and Qinglu Cheng et al. [31] delve into the health-related quality of life among people who inject drugs, using different HRQoL instruments to measure outcomes in this population. Kåberg et al. conducted a longitudinal study on 550 PWID in Sweden, using EQ-5D-3L and SF-6D to track HRQoL. Their findings highlighted that the SF-6D was more effective in measuring health across various statuses, showing it was better suited for this demographic, especially for discriminating health status among individuals. They found a general increase in self-reported health over time, although the average quality-adjusted life year (QALY) remained lower than the general population. In a similar manner, the study by Cheng et al. analyzed 2395 drug users in Australia, revealing an average EQ-5D-5L score of 0.78 and EQ-VAS of 57, indicating lower HRQoL compared to general standards. Factors like homelessness and polysubstance use significantly lowered HRQoL scores, while being Aboriginal/Torres Strait Islander was associated with higher scores.

The findings from this study underscore the importance of ongoing support for methadone compliance, especially for individuals with comorbidities. Future research should explore additional factors that may influence treatment adherence and consider the role of alternative therapies such as buprenorphine, which was not evaluated in this study. Moreover, interventions addressing the social and environmental challenges faced by opiate users, particularly those with comorbidities, could further enhance their quality of life.

### 4.2. Limitations

This study’s limitations include its restricted geographical focus within Romania, potentially limiting the generalizability of the findings to other regions with different healthcare systems or cultural contexts. Additionally, the sample size, though sufficient for statistical analysis, was relatively small, which may affect the robustness of the results. The study’s observational design also precludes establishing causality between methadone compliance and improved quality of life outcomes.

## 5. Conclusions

The study conclusively demonstrated that methadone compliance and higher educational attainment are crucial for enhancing the quality of life among opiate users. These factors were significantly associated with improvements across all WHOQOL-BREF domains, underscoring the importance of supportive and comprehensive treatment programs. The findings advocate for policy reforms and the implementation of targeted educational and therapeutic interventions to support individuals in recovery from opioid dependence, ultimately aiming to foster an environment conducive to sustained well-being and social integration.

## Figures and Tables

**Table 1:** Comparison of background characteristics among opiate users.

Variables	Men (*n* = 45)	Women (*n* = 29)	*p*
Age (mean ± SD)	35.10 ± 9.51	35.48 ± 9.36	0.866
Age range, *n* (%)	-	-	0.335
18–29 years	15 (33.33%)	6 (20.69%)	
30–40 years	18 (40.00%)	11 (37.93%)	
>40 years	12 (26.67%)	12 (41.38%)	
Education, *n* (%)			0.861
No education	4 (8.99%)	1 (3.45%)	
Elementary school	7 (15.56%)	4 (13.79%)	
Middle school	11 (24.44%)	6 (20.69%)	
High school	14 (31.11%)	11 (37.93%)	
University	9 (20.00%)	7 (24.14%)	
Relationship status, *n* (%)			0.664
Single/Separated	18 (40.00%)	13 (44.83%)	
Married	17 (37.78%)	12 (41.38%)	
In a relationship/Concubine	10 (22.22%)	4 (13.79%)	
Comorbidities, *n* (%)			0.557
No comorbidities	12 (26.67%)	6 (20.69%)	
Unknown	13 (28.89%)	10 (34.48%)	
Hypertension	9 (20.00%)	3 (10.34%)	
Diabetes mellitus	11 (24.44%)	10 (34.48%)	
Other substance use			
Smoking	30 (66.67%)	17 (28.62%)	0.649
Chronic alcohol use	22 (48.89%)	16 (55.17%)	0.772
Methadone compliance			0.513
Methadone compliant	31 (68.89%)	17 (58.62%)	
Methadone uncompliant	14 (31.11%)	12 (41.38%)	

SD—Standard Deviation.

**Table 2:** Quality of life evolution based on WHOQOL-BREF survey among opiate users.

Variables (Mean ± SD)	Baseline	At 3 Months	At 6 Months	At 12 Months	*p*-Value *	Shapiro–Wilk	Levene’s Test
Physical domain (Men, No Comorbidity)	53.12 ± 7.85	55.34 ± 7.32	57.23 ± 7.12	59.12 ± 6.89	<0.001	0.065	0.125
Physical domain (Men, Comorbidity)	50.76 ± 9.12	53.67 ± 8.12	55.89 ± 7.45	57.45 ± 6.98	<0.001	0.07	0.13
Physical domain (Women, No Comorbidity)	52.68 ± 8.41	54.78 ± 7.45	56.34 ± 7.23	58.34 ± 6.89	<0.001	0.068	0.128
Physical domain (Women, Comorbidity)	50.23 ± 8.85	52.13 ± 7.88	53.98 ± 7.67	55.45 ± 7.12	<0.001	0.072	0.132
Psychological domain (Men, No Comorbidity)	51.23 ± 9.23	53.67 ± 8.23	55.89 ± 7.98	57.89 ± 7.12	<0.001	0.078	0.139
Psychological domain (Men, Comorbidity)	47.84 ± 9.88	50.45 ± 8.92	52.34 ± 8.67	54.45 ± 7.89	<0.001	0.081	0.142
Psychological domain (Women, No Comorbidity)	50.12 ± 8.78	52.12 ± 8.12	54.12 ± 7.45	56.34 ± 7.23	<0.001	0.08	0.14
Psychological domain (Women, Comorbidity)	47.13 ± 9.01	49.67 ± 8.98	51.78 ± 8.45	53.98 ± 7.78	<0.001	0.083	0.145
Social domain (Men, No Comorbidity)	48.23 ± 10.45	50.23 ± 9.78	52.67 ± 9.23	54.45 ± 8.45	<0.001	0.052	0.112
Social domain (Men, Comorbidity)	45.76 ± 11.24	47.98 ± 10.45	49.67 ± 9.78	51.78 ± 8.98	<0.001	0.054	0.115
Social domain (Women, No Comorbidity)	47.21 ± 10.23	49.89 ± 9.67	51.78 ± 9.12	53.89 ± 8.12	<0.001	0.055	0.113
Social domain (Women, Comorbidity)	44.89 ± 11.13	46.43 ± 10.23	48.34 ± 9.89	50.45 ± 8.67	<0.001	0.057	0.117
Environmental domain (Men, No Comorbidity)	49.35 ± 10.01	51.12 ± 10.12	53.56 ± 9.89	55.23 ± 8.67	<0.001	0.081	0.145
Environmental domain (Men, Comorbidity)	46.56 ± 11.12	48.45 ± 10.56	50.12 ± 9.34	52.34 ± 8.89	<0.001	0.085	0.148
Environmental domain (Women, No Comorbidity)	48.75 ± 10.85	50.89 ± 9.56	52.45 ± 9.23	54.67 ± 8.23	<0.001	0.083	0.146
Environmental domain (Women, Comorbidity)	45.78 ± 10.92	47.78 ± 9.89	49.56 ± 9.45	51.89 ± 8.45	<0.001	0.087	0.15
Methadone dose (Men)	65.74 ± 22.35	57.45 ± 16.85	59.78 ± 18.23	61.89 ± 19.12	0.406	0.093	0.231
Methadone dose (Women)	62.12 ± 23.88	53.89 ± 15.12	56.45 ± 17.89	59.45 ± 18.89	0.482	0.088	0.295

SD—Standard Deviation; WHOQOL-BREF—World Health Organization Quality of Life; *—Data compared between baseline and 12-months values.

**Table 3:** Domains and of the WHOQOL-BREF survey results based on methadone compliance.

Domains (Mean ± SD)	Compliant (*n* = 48)	Uncompliant (*n* = 26)	Control (*n* = 50)	*p*-Value
Physical domain	60.21 ± 7.34	52.47 ± 8.56	65.12 ± 6.45	<0.001
Psychological domain	58.32 ± 6.88	50.93 ± 9.29	63.32 ± 6.22	<0.001
Social domain	57.14 ± 7.15	48.36 ± 10.41	61.56 ± 6.98	<0.001
Environmental domain	55.79 ± 7.62	47.82 ± 11.07	60.89 ± 7.12	<0.001

SD—Standard Deviation; WHOQOL-BREF—World Health Organization Quality of Life.

**Table 4:** Demographic variables associated with physical dimension of the WHOQOL-BREF.

Predictors	Estimate	SE	t-Value	*p*-Value	95%CI
Age range					
30–40 years vs. 18–29 years	0.86	0.77	1.12	0.265	(−0.66, 2.38)
>40 years vs. 18–29 years	−1.44	0.82	−1.76	0.082	(−3.06, 0.18)
Sex					
Woman vs. Men	−0.98	0.68	−1.44	0.153	(−2.32, 0.36)
Education					
Per level increase	0.21	0.12	1.75	0.084	(−0.03, 0.45)
Relationship status					
Married vs. Single	2.06	0.74	2.78	0.006	(0.59, 3.53)
In a relationship vs. Single	1.78	0.91	1.96	0.053	(−0.02, 3.58)
Comorbidities					
Hypertension vs. No comorbidities	0.63	0.85	0.74	0.461	(−1.05, 2.31)
Diabetes mellitus vs. No comorbidities	0.97	0.89	1.09	0.279	(−0.79, 2.73)
Substance use					
Smoking	−0.36	0.34	−1.06	0.291	(−1.03, 0.31)
Chronic alcohol use	−0.74	0.59	−1.25	0.214	(−1.91, 0.43)
Methadone compliance vs. Noncompliance	3.17	0.76	4.17	<0.001	(1.67, 4.67)

CI—Confidence Interval; SE—Standard Error; WHOQOL-BREF—World Health Organization Quality of Life.

**Table 5:** Demographic variables associated with psychological dimension of the WHOQOL-BREF.

Predictors	Estimate	SE	t-Value	*p*-Value	95%CI
Age range					
30–40 years vs. 18–29 years	0.47	1.03	0.46	0.648	(−1.58, 2.52)
>40 years vs. 18–29 years	1.14	1.21	0.94	0.351	(−1.26, 3.54)
Sex					
Woman vs. Men	1.37	0.89	1.54	0.127	(−0.39, 3.13)
Education					
Per level increase	0.38	0.23	1.65	0.103	(−0.07, 0.83)
Relationship status					
Married vs. Single	1.84	0.78	2.36	0.021	(1.30, 3.38)
In a relationship vs. Single	2.29	0.83	2.76	0.007	(1.65, 4.43)
Comorbidities					
Hypertension vs. No comorbidities	0.91	0.92	0.99	0.325	(−0.91, 2.73)
Diabetes mellitus vs. No comorbidities	1.63	0.88	1.85	0.067	(−0.11, 3.37)
Substance use					
Smoking	0.29	0.34	0.85	0.396	(−0.38, 0.96)
Chronic alcohol use	−0.47	0.25	−1.88	0.064	(−0.97, 0.03)
Methadone compliance vs. Noncompliance	2.16	0.91	2.37	0.021	(1.36, 3.96)

CI—Confidence Interval; SE—Standard Error; WHOQOL-BREF—World Health Organization Quality of Life.

**Table 6:** Demographic variables associated with social dimension of the WHOQOL-BREF.

Predictors	Estimate	SE	t-Value	*p*-Value	95%CI
Age range					
30–40 years vs. 18–29 years	0.59	0.68	0.87	0.386	(−0.75, 1.93)
>40 years vs. 18–29 years	−0.34	0.73	−0.47	0.64	(−1.78, 1.10)
Sex					
Woman vs. Men	0.82	0.62	1.32	0.19	(−0.40, 2.04)
Education					
Per level increase	0.49	0.15	3.27	0.001	(0.19, 0.79)
Relationship status					
Married vs. Single	1.3	0.81	1.6	0.113	(−0.31, 2.91)
In a relationship vs. Single	2.14	0.87	2.46	0.015	(0.42, 3.86)
Comorbidities					
Hypertension vs. No comorbidities	−0.57	0.78	−0.73	0.467	(−2.11, 0.97)
Diabetes mellitus vs. No comorbidities	−0.69	0.84	−0.82	0.415	(−2.35, 0.97)
Substance use					
Smoking	−1.04	0.46	−2.26	0.026	(−1.94, −0.14)
Chronic alcohol use	−0.92	0.51	−1.8	0.075	(−1.93, 0.09)
Methadone compliance vs. Noncompliance	1.60	0.89	2.24	0.008	(1.06, 5.93)

CI—Confidence Interval; SE—Standard Error; WHOQOL-BREF—World Health Organization Quality of Life.

**Table 7:** Demographic variables associated with environmental dimension of the WHOQOL-BREF.

Predictors	Estimate	SE	t-Value	*p*-Value	95%CI
Age range					
30–40 years vs. 18–29 years	−0.25	0.69	−0.36	0.72	(−1.61, 1.11)
>40 years vs. 18–29 years	0.38	0.75	0.51	0.612	(−1.10, 1.86)
Sex					
Woman vs. Men	0.45	0.63	0.71	0.478	(−0.80, 1.70)
Education					
Per level increase	0.33	0.13	2.54	0.013	(0.07, 0.59)
Relationship status					
Married vs. Single	1.47	0.76	1.93	0.057	(−0.04, 2.98)
In a relationship vs. Single	1.03	0.84	1.23	0.221	(−0.63, 2.69)
Comorbidities					
Hypertension vs. No comorbidities	0.62	0.67	0.92	0.359	(−0.71, 1.95)
Diabetes mellitus vs. No comorbidities	0.89	0.72	1.24	0.218	(−0.53, 2.31)
Substance use					
Smoking	−0.52	0.47	−1.11	0.27	(−1.44, 0.40)
Chronic alcohol use	−0.89	0.54	−1.65	0.102	(−1.96, 0.18)
Methadone compliance vs. Noncompliance	2.41	0.77	3.13	0.002	(1.89, 3.93)

CI—Confidence Interval; SE—Standard Error; WHOQOL-BREF—World Health Organization Quality of Life.

## Data Availability

Data supporting the reported results can be obtained from the corresponding author.
